# Pathohistological investigation of osteochondral tissue obtained during total knee arthroplasty after osteochondral autologous transfer: a case report

**DOI:** 10.1186/s13104-017-2513-0

**Published:** 2017-06-06

**Authors:** Momoko Tanima-Nagai, Hideto Harada, Tomoki Aoyama, Shoki Yamaguchi, Akira Ito, Junichi Tajino, Hirotaka Iijima, Xiankai Zhang, Hiroshi Kuroki, Masahiko Kobayashi

**Affiliations:** 1 Congenital Anomaly Research Center, Kyoto University Graduate School of Medicine, Konoe-cho, Yoshida, Sakyo-ku, Kyoto, 606-8501 Japan; 2 Department of Orthopedic Surgery, Kyoto Katsura Hospital, 17 Hirao-cho, Yamada, Nishikyo-ku, Kyoto, 615-8256 Japan; 3 Department of Physical Therapy, Human Health Sciences, Graduate School of Medicine, Kyoto University, 53, Kawahara-cho, Shogoin, Sakyo-ku, Kyoto, 606-8507 Japan; 4 Department of Physical Therapy, School of Nursing and Rehabilitation Sciences at Odawara, International University of Health and Welfare, 1-2-25 Shiroyama, Odawara, Kanagawa 250-8588 Japan; 5 Department of Orthopedic Surgery, Knee/Shoulder Surgery & Sports Medicine, Kyoto Shimogamo Hospital, 17 Higashimorigamae-cho, Shimogamo, Sakyo-ku, Kyoto, 606-0866 Japan

**Keywords:** Osteochondral autologous transfer, Pathohistology, Bone marrow edema, Case report

## Abstract

**Background:**

Osteochondral autologous transfer is one of the repair techniques for cartilage defects of knee with promising knee function recovery. There are no reports including histopathological images concerning human osteochondral tissue after osteochondral autologous transfer. This is the first report to present pathohistological findings of transplanted plugs and host tissues extracted from the human body 3 years after osteochondral autologous transfer. This study aimed to explore the cause factor of chronic pain using histological techniques.

**Case presentation:**

A 67-year-old Japanese man presented with adjusted total knee arthroplasty 3 years after osteochondral autologous transfer. Although in pain, arthroscopic assessment was not severe. The specimens which was gained during total knee arthroplasty were investigated in gross and microscopically using immunohistochemical staining technic. Histological examination revealed that the gap between grafted plugs and host osteochondral tissues was filled with fibrous tissue that stained positive for type I collagen. A degenerative change and some neovascularity were observed in the regenerated tissue and host trabecular bone. Furthermore, cysts and bone marrow edema were observed.

**Conclusion:**

Our data suggests that the host osteochondral morbidity around grafted plugs might be related to chronical pain and revision surgery.

## Background

Osteochondral autologous transfer (OAT) is a repair technique for cartilage defects with good prognosis [1, 2]. There have been numerous reports about clinical outcomes after OAT in humans [1–5], and concerning the histopathology in animal models [6–9]. In contrast, there have been few reports about the histopathology and morbidity of the implanted plugs in humans due to ethical reasons. The purpose of this study was to histologically explore the factors leading to chronic pain and revision surgery after OAT. Histological signs would provide information that cannot be obtained by surface observations, which would be helpful in understanding the pathology and in improving the operative technique (Approval an ethics panel no: Kyoto University C186).

## Case presentation

**Fig. 1:**
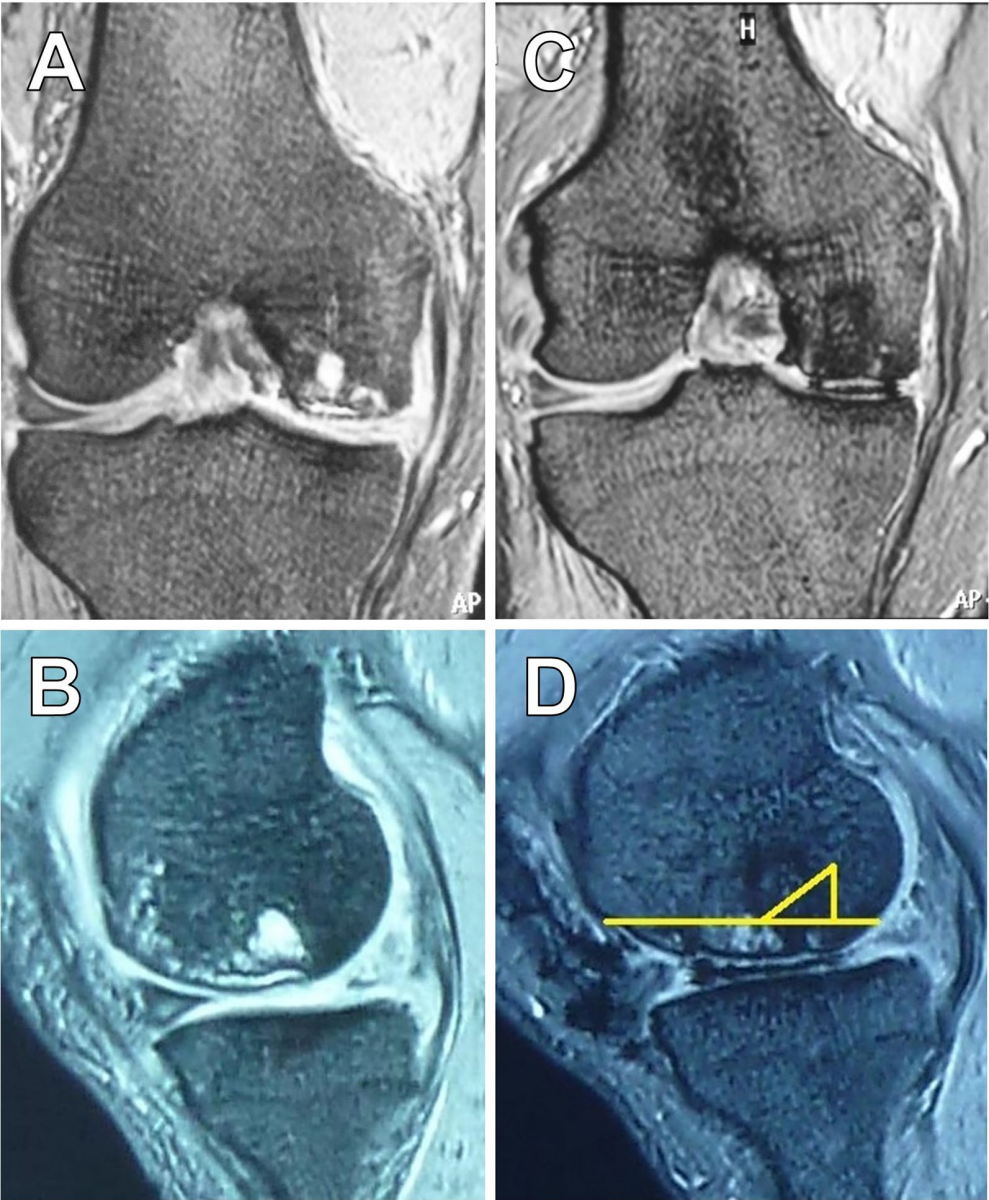
Magnetic resonance image of pre- and post-osteochondral autologous transfer operation. Low intensity on T2-weighted images of the right medial femoral condyle (**A**, **C** coronal plane; **B**, **D** sagittal plane). The images were taken before osteochondral autologous transfer (**A**, **B**) and before total knee arthroplasty (**C**, **D**). *Yellow lines* show the resection line of two parts of the tissue

**Fig. 2:**
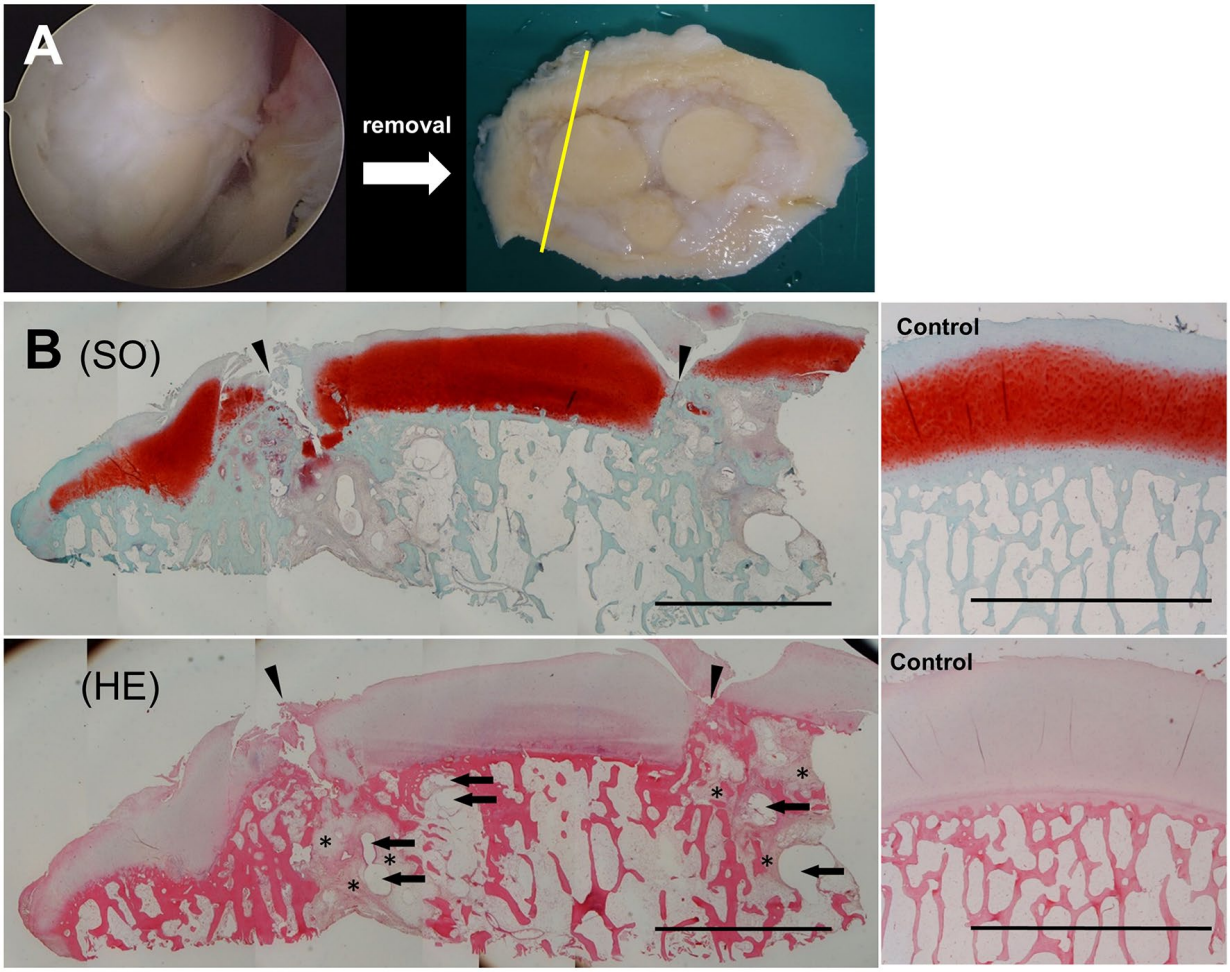
Arthroscopic images after osteochondral autologous transfer (OAT), and macro and micro observation images of isolated tissue after total knee arthroplasty (TKA). **A** At 1.5 years after OAT, good prognosis of the transferred grafts was confirmed by arthroscopy (*left side*). The osteochondral tissue contained inserted plugs that were discarded during TKA. The surface of the tissue was almost smooth (*right side*). The *yellow line* indicates the section area of (**B**). **B** Histological images with safranin-O staining (*upper*; SO) and hematoxylin–eosin staining (*lower*; H–E) show that the cartilage surface was not smooth between the host and plug cartilage. There were some cracks in the regenerated fibrous cartilage that reached bone marrow region. *Arrowhead* indicates the site of transferred plugs. *Asterisk* indicates bone marrow edema. *Arrows* indicate subchondral bone cyst. The control tissue shows the smooth surface of cartilage and strong staining intensity of SO. *Scale bars* indicate 5 mm

A 67-year-old Japanese man (body mass index, BMI = 27.7) presented to the hospital with right knee pain diagnosed as spontaneous osteonecrosis of the knee and was treated conservatively. Three years later, OAT was performed because of increased pain and cyst formation observed in the subchondral area by magnetic resonance imaging. Before OAT, T2-weighted images showed a wide area of low intensity in the right medial femoral condyle (MFC) that reached the bone marrow region (Fig. 1A, B); the Lysholm score was 49/100. The degenerated region measured 25 × 20 mm in the weight-bearing area of the MFC and was considered a defect after OAT. A total of three grafts, 10 mm or 6 mm in diameter with a depth 16 mm, were harvested from the non-weight-bearing portion of the patella groove and were transferred to the defect area. At 1.5 years after OAT, we confirmed that the International Cartilage Repair Society score was grade II and the Lysholm score was 85/100, although the pain remained a little. Total knee arthroplasty (TKA) was performed 3 years after OAT because of chronic and intense pain. T2-weighted images showed a wide range of osteonecrosis with high signal intensity (Fig. 1C, D). Two parts of osteochondral tissue were isolated so that they could be discarded during TKA; the osteochondral tissue containing implanted plugs (Fig. 2A) and the host trabecular bone located deeper than the engrafted tissue. Isolated tissues were observed macroscopically, fixed, decalcified, and embedded in paraffin. Then, 6-μm sections were stained with hematoxylin–eosin (H–E) and safranin-O (SO). The expressions of type I and type II collagen and vascular endothelial growth factor (VEGF) were analyzed immunohistochemically. We also investigated a comparative control tissue extracted from the normal area of a middle-aged man who underwent TKA.

**Fig. 3:**
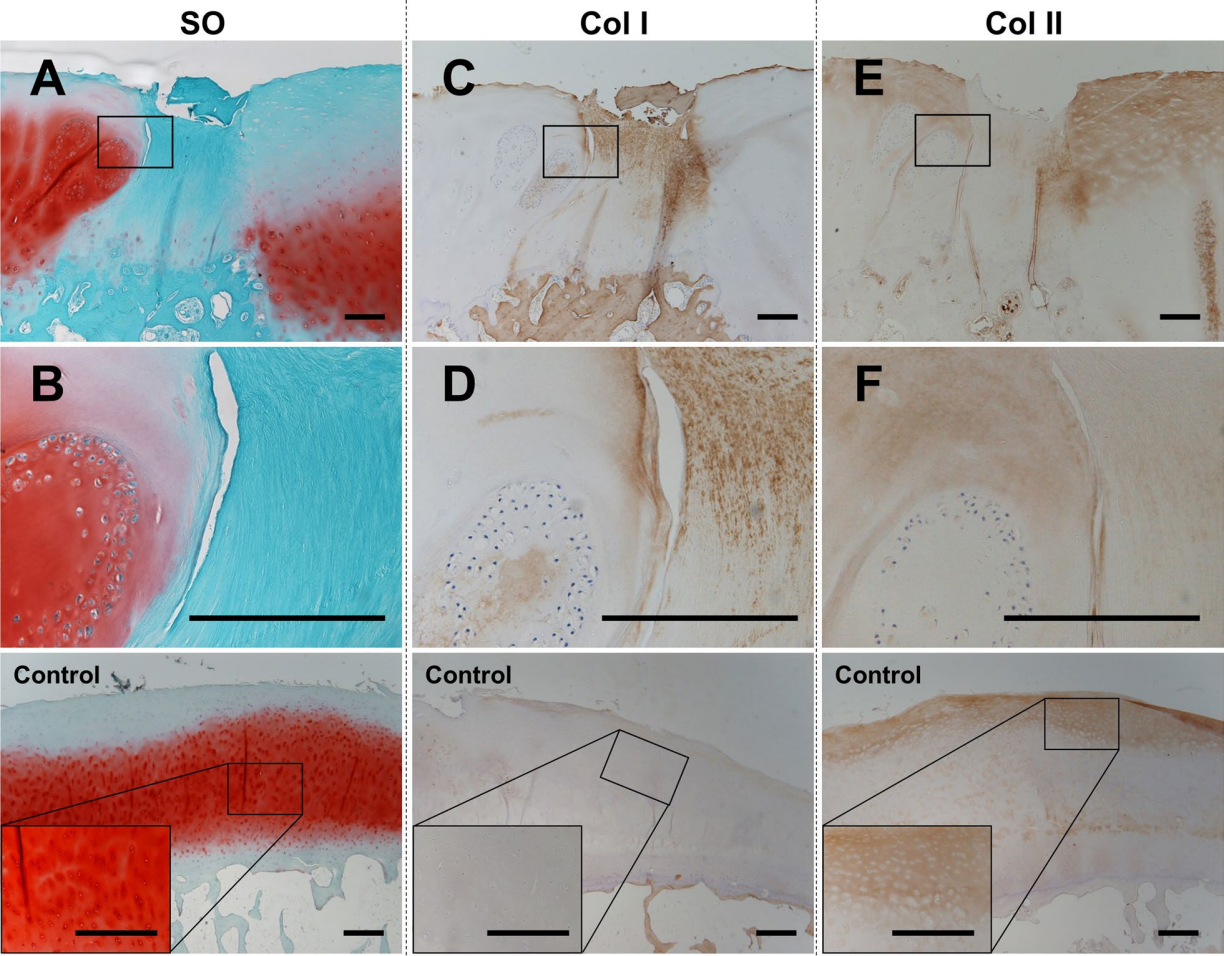
Histological and immunohistological stained images of repaired tissue between plugs and control tissue. Repair tissue between plugs with safranin-O (**A**, **B**), type I collagen (Col I; **C**, **D**), and type II collagen (Col II; **E**, **F**) show that the repaired tissue was not hyaline cartilage because plug cartilage and cluster cells existed in the cartilage adjacent to the repaired tissue. **A**, **C**, **E** Low magnification (×20). **B**, **D**, **F** High magnification images of quadrilateral area in above images (×200). *Scale bars* indicate 600 μm

**Fig. 4:**
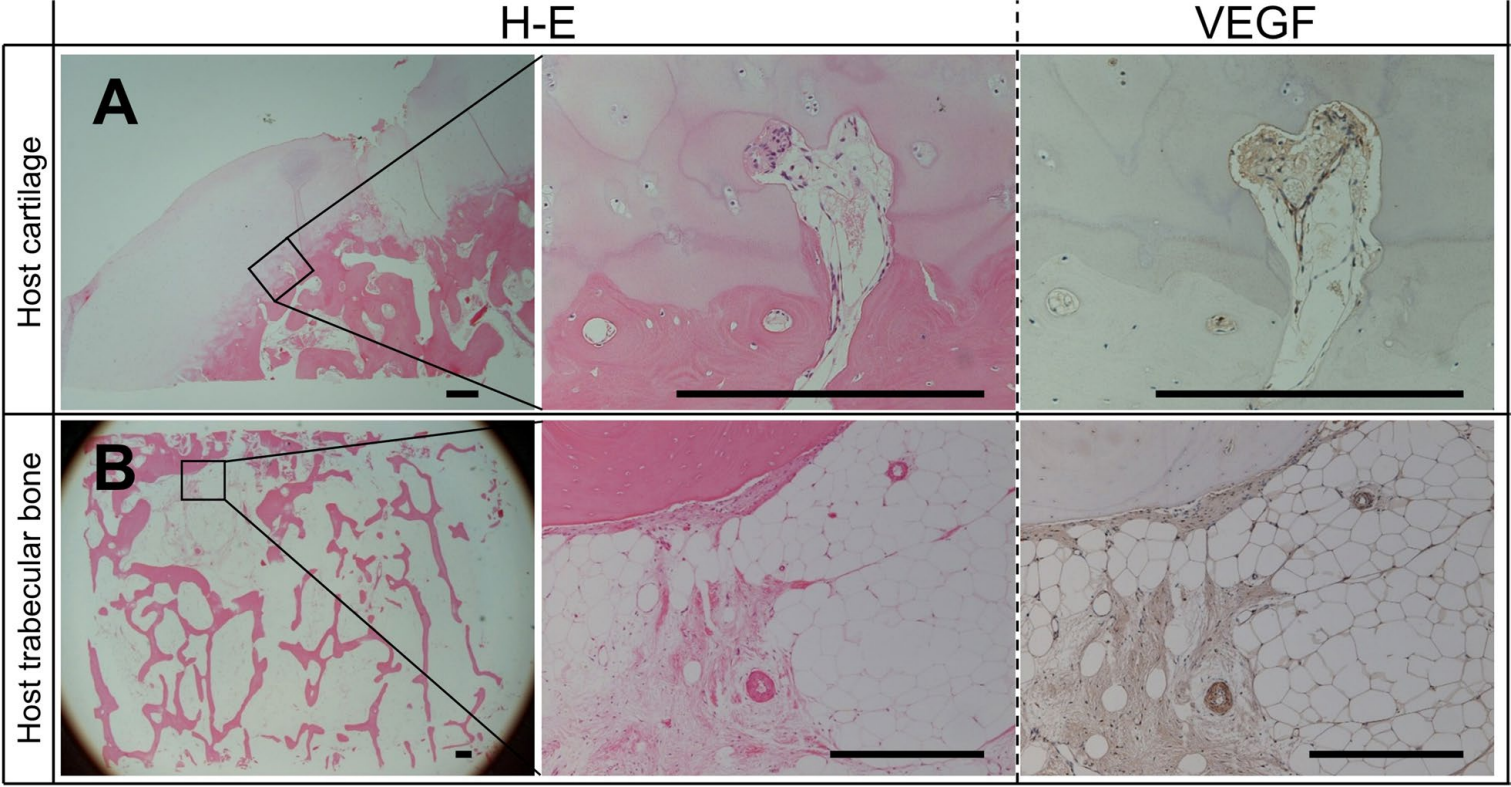
The results of vascular endothelial growth factor (VEGF) expression and hematoxylin–eosin (H–E) staining at host cartilage and host trabecular bone. VEGF-positive cells and microvascular were often confirmed in the subchondral region connected to the cartilage region (**A**: *upper lane*) and in the host trabecular bone region (**B**: *lower lane*). *Scale bars* indicate 200 μm

On macroscopic observation, the tissue of our patient was filled with regenerated tissue among all plugs and host tissue. The surface was almost smooth (Fig. 2A). On microscopic observation, strong SO staining was observed in the engrafted plug, host cartilage, and control tissue (Fig. 2B). The gap between plugs is usually filled with regenerated fibrous tissue with less SO and type II collagen staining intensity. Instead, type I collagen staining intensity was denser than that for the plug, host cartilage, or control tissue (Fig. 3). There were some cracks in the regenerated fibrous tissue that reached the bone marrow region. Bone marrow edema (BME) or cyst formation within the microvasculature was confirmed mostly in the bone marrow region and these findings were not observed in control tissue (Fig. 2B). Neovascularity indicated by VEGF staining was frequently observed in subchondral area connected to the bone marrow spaces and deep subchondral area in the plug and in the host trabecular bone near the BME (Fig. 4).

## Discussion

Some systematic reviews of OAT have reported that risk factors for failure are age <30 years, two or more previous surgeries, and defect area >4–5 cm^2^ [1, 10]. Enabling a better healing capability of the young may lead to a better prognosis after OAT [4]. Aging and being overweight are also important risk factors for osteochondral complex degeneration such as osteoarthritis [11]. Being male and overweight (as indicated by BMI) to be the cause of OAT failure in our patient might be speculation; the influence of gender and BMI on OAT outcome is not clear [12].

In this study, we observed osteochondral plugs and host tissue obtained from a patient with spontaneous osteonecrosis diagnosed before OAT surgery. Some cracks reached the subchondral bone in the regenerated fibrocartilage; nevertheless, it was filled with regenerated tissue on macroscopic and arthroscopic observations. Furthermore, BME existed at the subchondral bone and host trabecular bone. A study regarding osteonecrosis reported that osteoporotic bone is more susceptible to microfracture and could be induced by increased or repeated stress in the subchondral bone plate and around the tissue [13]. Microfractures lead to fluid accumulation [14] and intraosseous edema, which cause increased pressure within the marrow cavity and increased vascular compromise of the subchondral bone or worsening of edema, finally resulting in necrosis or cyst [13]. The underlying marrow is rich in nociceptive fibers [15], explaining the close relationship between BME and intraosseous hypertension with pain [16, 17]. The instability of the morbid region created abnormal mechanical stress, thus producing catabolic pathways in an osteochondral complex [11, 18]. It is suggested that the existence of a morbid region weakened by mechanical stress in host osteochondral tissue affects cyst and BME formation, leading to the patient’s chronic intense pain.

## Conclusion

This study showed the pathohistological findings of transplanted plugs and host tissues extracted from the human body 3 years after OAT. The large extent of the degenerated host area might cause deterioration in the transplanted and regenerative osteochondral tissue, and chronic pain, leading to revision surgery. There is a possibility that removal of the morbid host region and partial bone remodeling could lead to good functional outcomes.

## Abbreviations

OAT: osteochondral autologous transfer
BMI: body mass index
MFC: medial femoral condyle
TKA: total knee arthroplasty
H–E: hematoxylin–eosin
SO: safranin-O
VEGF: vascular endothelial growth factor
BME: bone marrow edema
